# Priming with a gonadotropin-releasing hormone agonist before immature oocyte retrieval may improve maturity of oocytes and outcome in *in vitro* maturation (IVM) cycle: a case report

**DOI:** 10.1186/s13256-021-02706-8

**Published:** 2021-03-27

**Authors:** A. Smirnova, M. Anshina, E. Shalom Paz, A. Ellenbogen

**Affiliations:** 1 IVF and Genetics Center “FertiMed”, Moscow, Russia; 2 Department of Endocrinology, Postgraduate Medical Faculty, IVF Unit, Russian University of People’s Friendship, Moscow, Russia; 3 Hillel Yaffe Medical Center, Hadera, Israel; 4 Rappaport School of Medicine, Technion-Israel Institute of Technology, Haifa, Israel

**Keywords:** GnRH agonist priming, *In vitro* maturation, Oocyte, IVF, *In vitro* fertilization, Case report

## Abstract

**Background:**

The concept of using a gonadotropin-releasing hormone agonist (GnRH-a) instead of human chorionic gonadotropin for triggering ovulation in patients treated with an antagonist protocol for *in vitro* fertilization (IVF) has become a routine clinical practice. It may promote oocyte nuclear maturation, resumption of meiosis and cumulus expansion. It seems that this attempt could be beneficial in an *in vitro* maturation (IVM) oocyte cycle performed for polycystic ovarian syndrome as well as for other indications such as urgent fertility preservation in patients with malignancies or unusual indications.

**Case presentation:**

We present the case of a Caucasian patient who needed fertility preservation when routine natural IVF treatment did not yield oocyte retrieval, followed by three IVM cycles, priming ovulation with a GnRH-a. In total, 12 oocytes were obtained, all matured 4.5 hours after incubation in maturation media. The fertilization rate after intracytoplasmic sperm injection was 83%. Six good-quality embryos were vitrified.

**Conclusions:**

It seems that triggering with a GnRH-a in selected cases may replace human chorionic gonadotropin in IVM of oocytes and could be highly beneficial in terms of obtaining high-grade embryos and possible pregnancy.

## Background

The conception of *in vitro* maturation (IVM) of oocytes was first reported in 1935 [1]. Experiments were described in which ova taken from fallopian tubes at various intervals after fertile mating were cultured *in vitro*. A successful IVM cycle in humans using immature oocytes retrieved from antral follicles was reported later, followed by the first delivery from IVM of oocytes recovered from patients with untreated polycystic ovarian syndrome (PCOS) [2]. As early as 1994, PCOS patients were the first natural candidates for IVM [2]. Recently, the range of clinical applications of IVM has been emphasized as an additional opportunity for germ cell preservation in women suffering from cancer [3]. Novel radio- and chemotherapy treatments can significantly improve the prognosis of these patients but, unfortunately, have major effects on ovarian function, often leading to premature ovarian failure. Technically, immature oocytes collected from antral follicles in the absence of gonadotropin administration may be cryopreserved before or after maturation *in vitro*. Therefore, IVM may represent a suitable opportunity for recovery of oocytes intended for cryopreservation in cases where tumor estrogen sensitivity and/or urgency to commence therapy prevent a full controlled ovarian stimulation [3]. However, different studies have reported that implantation and pregnancy rates after IVM were lower than with traditional *in vitro* fertilization (IVF), and varied from 5 to 22% [4]. It is possible that in an IVM cycle, although nuclear maturation of metaphase II (MII) oocytes obtained after maturation in proper medium progresses normally, the cytoplasmic maturation is delayed, resulting in poorer embryonic development, implantation and pregnancy rates [5].

Human chorionic gonadotropin (hCG) has been the gold standard for ovulation induction as a surrogate for the mid-cycle luteinizing hormone (LH) surge. In recent years, the concept of using a gonadotropin-releasing hormone agonist (GnRH-a) for triggering ovulation was reintroduced among patients treated by an antagonist protocol for IVF. Interestingly, it was suggested that it may promote oocyte nuclear maturation, that is, resumption of meiosis and cumulus expansion [6]. It was recently suggested that triggering final oocyte maturation with GnRH-a versus hCG in IVM cycles among breast cancer patients undergoing fertility preservation could be beneficial, as evidenced by the number of mature oocytes and cryopreserved embryos [7]. Therefore, it seems that this case report may add to existing knowledge regarding the beneficial effect of GnRh-a as a triggering approach in consecutive IVM cycles performed for fertility preservation in patients with malignancies or uncommon indications.

## Case presentation

A 38-year-old Caucasian housewife was referred to the IVF and Reproductive Genetics Center, Moscow, for fertility preservation. She and her family lived in a small nearby town with normal ecological conditions. There was no history of serious illness in her family. She used barrier contraception and had never been pregnant. She had never smoked or consumed alcohol.

She had regular, 25–28-day menstrual periods. Her past history revealed that she had undergone laparoscopy and unilateral right adnexectomy due to a large papillary mucinous cystadenoma, 10 cm in diameter. Ten years later, a new cyst, 4 cm in diameter, was diagnosed on the contralateral ovary. Laparotomy with partial ovarian resection was performed. Histological examination again revealed mucinous cystadenoma. She was otherwise healthy, height 166 cm and body mass index (BMI) 24.7.

On admission, physical and neurological examination yielded no pathological findings. Gynecological examination was unremarkable. Blood pressure was 115/75 mmHg, resting heart rate 78 beats per minute, and temperature was normal. Full laboratory tests (complete blood count [CBC], basic, complete metabolic and coagulation panel) were in the normal range. Test results for HIV antibodies, hepatitis B antigen and antibodies, hepatitis C antibodies, Lues serology, toxoplasma and cytomegalic virus antigens and antibodies were negative. Polymerase chain reaction was negative for *human papillomavirus* (HPV). Pap smear test was normal. Her blood type was O, rhesus (Rh)-negative. Coombs test was negative. Her hormone profile on day 3 of the cycle was as follows: anti-Müllerian hormone (AMH) 0.35 ng/mL; follicle-stimulating hormone (FSH) 6.9 IU/L; LH 8.9 IU/L; prolactin 190 mIU/L; thyroid-stimulating hormone (TSH) 1.77 mIU/L; testosterone 0.77 nmol/L. Vaginal ultrasound (U/S) on day 7 of her cycle showed a normal sized uterus. Left ovary was 34×23×22 mm containing an 18×16 mm cyst and five small antral follicles. Her 30-year-old partner had normal sperm parameters. His routine serology tests were negative.

In accordance with Russian legislation, ovarian stimulation in women with ovarian tumors (even small) is prohibited. Therefore, a natural cycle IVF was commenced, but no oocyte was retrieved.

In order to improve results and obtain oocytes without stimulating the ovaries, we decided to conduct an IVM cycle. A total of three successive IVM attempts were performed.

## First IVM cycle

U/S monitoring was started on day 6 of the cycle. Endometrial thickness was 5 mm, and three antral follicles, 7.5 mm, 8.5 mm and 6 mm in diameter, were visualized on the left ovary. On day 8 of the cycle, the follicles were 8 mm, 9.5 mm and 7 mm, and triptorelin (Decapeptyl, Ferring GmbH, Kiel, Germany) 0.2 mg was administered subcutaneously to trigger oocyte maturation. Transvaginal U/S-guided follicle aspiration was performed 39 hours later using a 19G/17G single lumen needle (Swemed Sense, Vitrolife, Göteborg, Sweden) and a reduced aspiration pressure of 7.5 kPa. Follicular fluid was collected without flushing into 50 mL dishes with Oocyte Washing Medium (SAGE IVM media kit, Cooper Surgical, USA)**.** A total of three oocyte-cumulus complexes (COCs) were obtained. All COCs were cultured in maturation medium (SAGE IVM media kit) supplemented with FSH+LH (Merional, IBSA Institut Biochimique SA, Switzerland) for a final concentration of 75 mIU/mL. Retrieved oocytes were stripped, found in the MII stage and fertilized by intracytoplasmic sperm injection (ICSI) 6 hours after aspiration as described previously [8]. All oocytes were fertilized 18 hours after ICSI. Two top-quality embryos (8 cells, grade 1) and one good-quality embryo (8 cells, grade 2) were vitrified 72 hours after fertilization. No luteal phase support was given to the patient.

## Second IVM cycle

Ten days after commencement of the menstrual cycle, endometrial thickness was 6.5 mm, and five antral follicles 10, 10.5, 8, 6 and 6 mm in diameter were demonstrated. Triptorelin 0.2 mg was injected subcutaneously. Ovum pickup was performed 38.5 hours later.

Six COCs were obtained, cultured in maturation media, stripped and fertilized by ICSI 4.5 hours after pickup. Normal fertilization occurred in five oocytes; the sixth developed one pronucleus. One top-quality embryo (10 cells, grade 1) was vitrified 72 hours after ICSI. Other embryos were cultured until day 6; two poor-quality blastocysts were obtained and discarded.

## Third IVM attempt

On the next menstrual cycle, U/S performed on day 10 revealed endometrial thickness of 7 mm and four antral follicles 10, 8, 6 and 5 mm in diameter. Transvaginal follicle aspiration was performed 39 hours after subcutaneous injection of 0.2 mg triptorelin as described above. Three COCs were obtained, cultured in maturation media for 5 hours, stripped, found to be in MII stage and fertilized by ICSI. Two developed 2 pronuclei (2PN) 18 hours after fertilization. Two good-quality embryos were vitrified on day 3. All IVM cycles were well tolerated, with no side effects.

In summary, 12 COCs were obtained following IVM of small antral follicle aspiration. All oocytes were found to be mature (MII) about 4.5 hours after incubation in maturation media. The fertilization rate after ICSI was 10/12 (83%) and cleavage rate was 10/12 (83%). A total of six good-quality embryos were vitrified on day 3. The patient recently underwent uneventful cystectomy ruling out malignancy. Frozen embryo transfer is planned.

## Discussion

We report here for the first time that repeated IVM cycles with GnRH-a triggering were successful and resulted in the collection of six high-quality embryos.

The emerging technology of IVM and oocyte retrieval has recently become an additional option for fertility preservation. This procedure can be done without hormonal stimulation and within a short time frame, as oocytes are collected during the follicular phase or even luteal phase treatment with a reasonable number of harvested oocytes [9]. Therefore, in cases of patients with malignancies, especially when hormone treatment is contraindicated, and in those who must start chemotherapy without delay or in unusual cases, IVM might be a preferred option to preserve fertility.

Oktay *et al*. [10] were the first to report the use of a GnRH analog as an oocyte maturation trigger for women with breast cancer undergoing IVF, and found that the number of oocytes retrieved, maturation and fertilization rates, and the number of 2PN embryos were significantly higher than with hCG trigger. Similar results were reported in breast cancer patients undergoing IVM cycles for urgent fertility preservation [8]. Recently, Dahan *et al*. [11] described GnRH-a triggering in a modified natural IVF cycle in a patient with PCOS. In this approach, follicles were stimulated with gonadotropins for 3 to 5 days when they were small, and ovulation was triggered with a GnRH-a when the largest follicles were 10–12 mm in diameter. Many immature oocytes were retrieved, matured *in vitro* and subsequently developed to form blastocysts that resulted in a live birth.

The rationale for triggering with a GnRH-a instead of hCG, mainly in an IVM cycle, is the induction of FSH surge comparable to the surge of the natural cycle. This surge may promote the formation of LH receptors on granulose cells, enhancing LH activity, inducing plasminogen activator activity that enhances dissociation of oocytes from the follicle wall (therefore more immature oocytes can be obtained), maintaining the opening of the gap junction between the cumulus cells and oocyte (enhancing oocyte maturation) [12, 13], promoting cumulus expansion [14] and possibly promoting cytoplasmic maturation thought to be delayed in IVM cycle triggering with hCG before oocyte retrieval. There is evidence that the release of LH and FSH after single administration of GnRH-a in an IVF cycle is able to complete the final stage of follicular maturation, resulting in retrieval of fertilizable oocytes, with normal embryo development and pregnancy [6]. Therefore, the combined action of GNRH-a, FSH and LH may have a beneficial effect on oocyte maturation, mainly in an IVM cycle.

In our three IVM cycles, 12 oocytes were obtained. Interestingly, all oocytes (100%) were found to be mature (MII) after 4.5–5 hours of culture in maturation media, in contrast to results in IVM cycles after hCG triggering, where only 15.6% were found to be mature up to 6 hours after retrieval, versus 64.9% that matured *in vitro* after 6–48 hours [15]. Indeed, from a semantic point of view, it could be suggested that in our case, the mature eggs obtained a short time after retrieval, which gave rise to high-grade embryos with possible high implantation potential, are not "really" IVM oocytes. However, the most important point is the result of obtaining six high-quality embryos that were vitrified.

## Conclusions

To the best of our knowledge, this case report is one of the few reports in the literature of an unusual case involving GnRH-a priming before oocyte retrieval in an IVM protocol. Moreover, originally, we performed three successive cycles, accumulating high-quality embryos. Therefore, it seems that triggering ovulation with GnRH-a instead of hCG in an IVM cycle performed for fertility preservation or rare cases, as described herein, could be valuable in terms of obtaining higher-grade embryos, possibly due to more synchronous nuclear and cytoplasmic maturation.

The present report describes a new alternative for treating uncommon cases of infertility using consecutive repeated IVM cycles with GnRH-a triggering.

## Data Availability

Not applicable.

## Abbreviations

GnRH-a: Gonadotropin-releasing hormone agonist
IVM: *In vitro* maturation
PCOS: Polycystic ovarian syndrome
ICSI: Intracytoplasmic sperm injection
U/S: Ultrasound
hCG: Human chorionic gonadotropin
COCs: Oocyte-cumulus complexes
